# Interspecies evaluation of a physiologically based pharmacokinetic model to predict the biodistribution dynamics of dendritic nanoparticles

**DOI:** 10.1371/journal.pone.0285798

**Published:** 2023-05-17

**Authors:** Christina Vasalou, Joanna Harding, Rhys D. O. Jones, Niresh Hariparsad, Dermot F. McGinnity

**Affiliations:** 1 Oncology R&D, AstraZeneca, Boston, Massachusetts, United States of America; 2 Oncology R&D, AstraZeneca, Cambridge, United Kingdom; University of Veterinary and Animal Sciences, PAKISTAN

## Abstract

The exposure of a dendritic nanoparticle and its conjugated active pharmaceutical ingredient (API) was determined in mouse, rat and dog, with the aim of investigating interspecies differences facilitating clinical translation. Plasma area under the curves (AUCs) were found to be dose proportional across species, while dose normalized concentration time course profiles in plasma, liver and spleen were superimposable in mouse, rat and dog. A physiologically based pharmacokinetic (PBPK) model, previously developed for mouse, was evaluated as a suitable framework to prospectively capture concentration dynamics in rat and dog. The PBPK model, parameterized either by considering species-specific physiology or using alternate scaling methods such as allometry, was shown to capture exposure profiles across species. A sensitivity analysis highlighted API systemic clearance as a key parameter influencing released API levels. The PBPK model was utilized to simulate human exposure profiles, which overlaid dose-normalized data from mouse, rat and dog. The consistency in measured interspecies exposures as well as the capability of the PBPK model to simulate observed dynamics support its use as a powerful translational tool.

## Introduction

Physiologically Based Pharmacokinetic (PBPK) models serve as quantitative tools integrating anatomical and physiological components of an organism with metabolic and biodistribution processes of an administered drug within the body. PBPK models are comprised of compartments corresponding to individual tissues–which may include the liver, kidney, spleen, muscle, adipose tissues, gut and brain–connected by the circulating blood system [1, 2]. Tissues, which are not organs of interest or do not significantly contribute to the overall PK may be lumped into a remainder or “rest” compartment to simplify kinetics [3]. In general, PBPK models contain two sets of parameters: 1) ones that are descriptive of the physiology and anatomy of the body, such as tissue volumes and blood flow rates and 2) ones that are specific to the drug administered, such as the clearance rates or partition coefficients. Physiological parameters are well characterized across species with well-defined values reported in the literature [4, 5], while drug-specific parameters across species are typically estimated either via simple allometry or by scaling relevant in-vitro data (e.g. metabolic clearance rates of small molecules derived from hepatocyte stability data) [6, 7]. Within this context, the implementation of PBPK models to describe nanoparticle biodistribution dynamics requires the derivation of two separate parameters sets: one related to the nanocarrier and the other related to the API released over time.

Nanoparticles explored in this study were dendrimers: branched polymers consisting of concentric rings of monomer, known as generations, radially added to one or more reactive groups, known as the central core [8, 9]. Dendrimers contain covalently conjugated drugs, released in response to exogenous stimuli such as pH or temperature [10]. The sensitivity of the drug/dendrimer linker stability to various stimuli has been utilized when optimizing bioanalytical methods [11, 12], rendering possible the measurement of both nanoparticle-released API as well as total API concentrations, defined as the sum of released and dendrimer-conjugated API.

The objective of this work was the evaluation of a PBPK model, previously developed for mouse [12], as a suitable mathematical framework for capturing nanoparticle dynamics in rat, dog and ultimately human. This work focused on released and total API dynamics in plasma as well as liver and spleen, selected due to their involvement in the reticuloendothelial system (RES) responsible for nanoparticle detection and uptake ultimately facilitating their decomposition. Multispecies pharmacokinetic data and mathematical modeling investigating the disposition of nanoparticles are limited [6]. Additionally, owing to bioanalytical challenges, the majority of nanoparticles explored preclinically only measure nanocarrier levels or total drug concentrations [6, 13, 14]. This is one of the first studies investigating interspecies nanoparticle biodistribution differences, while also providing quantification and mathematical descriptions of both released and total drug states across plasma and relevant tissues towards the derivation of clinical exposure profiles.

## Results

### Nanoparticle pharmacokinetic and biodistribution data in mouse, rat and dog

**Fig 1:**
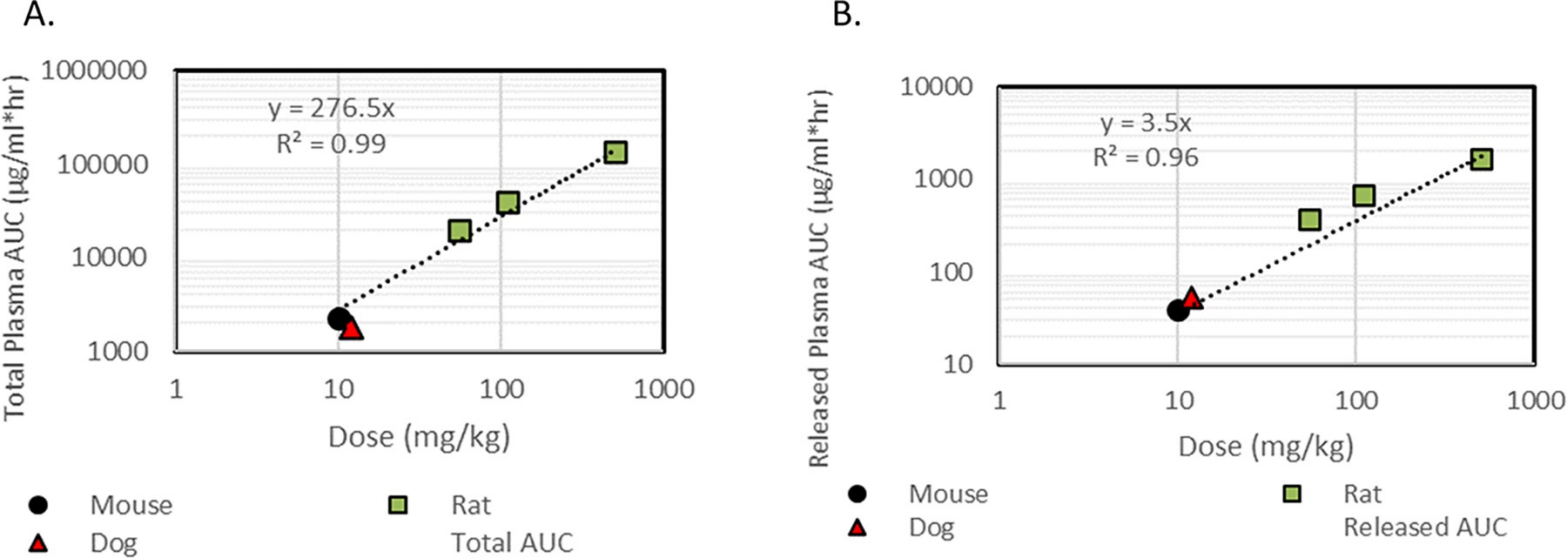
Total and Released API AUC is shown across mouse, rat and dog for increasing doses of API. The relationship between plasma AUC and dose follows a linear equation: AUC = 276.5*dose (mg/kg) for the total API (A) and AUC = 3.5 *dose(mg/kg) for the released API (B). Mouse is shown in black circles; rat in green squares; dog in red triangles.

A single intravenous (IV) dose of the nanoparticles was administered in mice, rats and dogs to investigate biodistribution trends across species. Concentration profiles were obtained from plasma, liver and spleen, measuring both released and total API. Total API was defined as the sum of nanoparticle-released and conjugated API. Investigated dose levels–defined as mg of API per Kg body weight–were 10 mg/kg in mouse administered as an IV-bolus, 55, 110 and 505 mg/kg in rat administered as 30-minute infusions and 12 mg/kg in dog also administered as 30-minute infusions. Total and released API AUCs, plasma clearance rates and half-lives were derived by implementing non-compartmental analysis (NCA) on plasma concentrations (Table 1). Dose normalized AUCs of total and released API were similar not only for rat, where tested doses ranged approximately ten-fold, but also across species. The relationship between both total and released API AUC versus dose is shown in Fig 1. In alignment with this observation, clearance rate and half-life values of total and released API were also found similar–approximately within 2-fold–across species and dose groups (Table 1).

**Table 1:** Total and released API AUC, clearance rates and half–lives across species and doses.

Species	Dose (mg/kg)	Total API Plasma AUC (μg/ml*h)	Total API half-life (h)	Total API plasma CL (L/h/kg)	Released API Plasma AUC (μg/ml*h)	Released API half-life (h)	Released API plasma CL (L/h/kg)
Mouse	10	2294	7.4	0.004	38.7	7.6	0.26
Rat	55	19593	6.8	0.003	373	11.5	0.15
Rat	110	40187	8	0.003	694	14	0.16
Rat	505	136989	15	0.004	1714	20.7	0.3
Dog	12	1812	7.8	0.007	56.3	7.9	0.21

**Fig 2:**
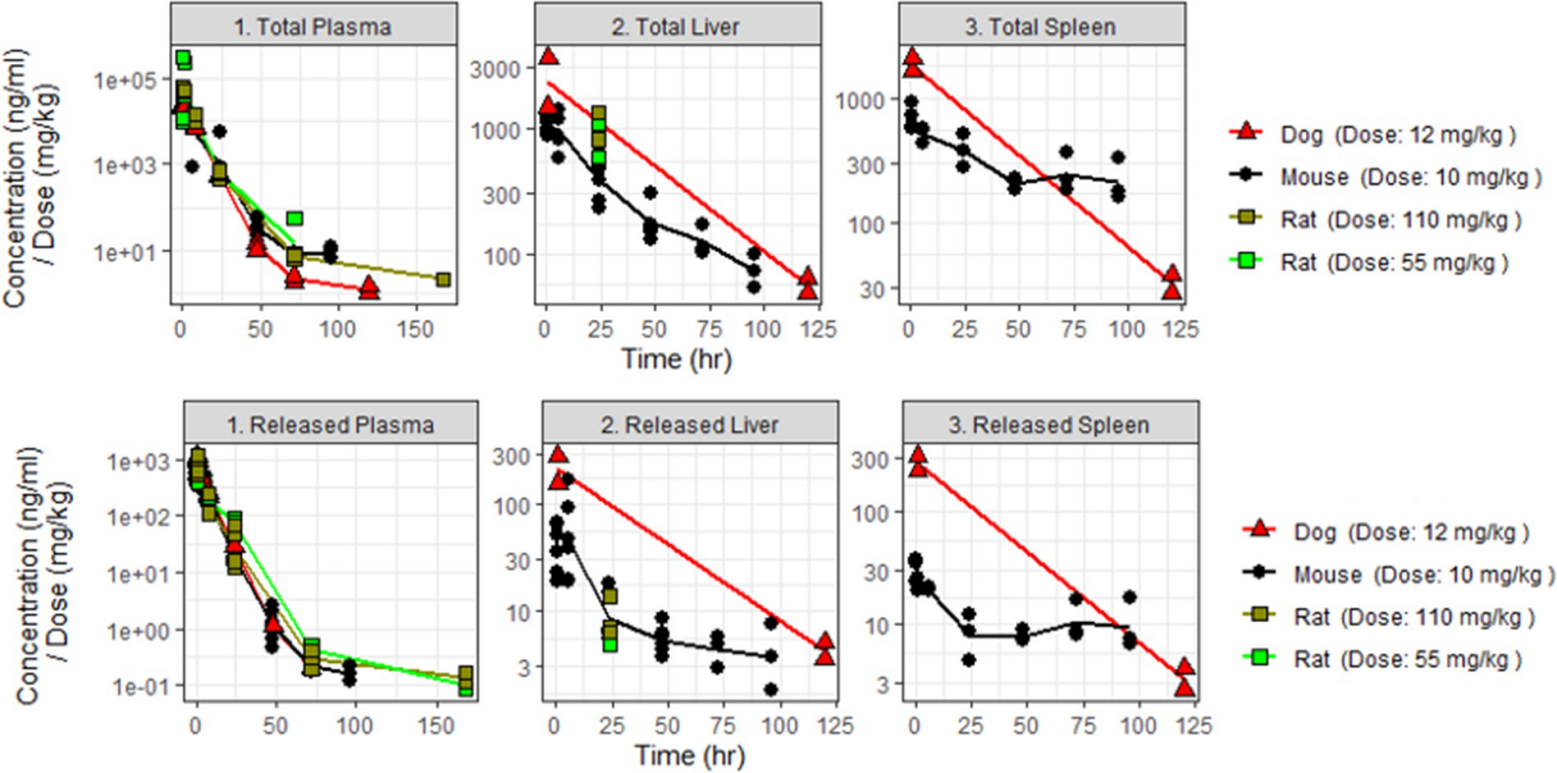
Dose–normalized concentration time course profiles of total (upper panels) and released API (lower panels). Solid lines represent the average data; closed shapes represent datapoints from each animal. Dose levels are defined as mg of API per Kg of body weight. Mouse is shown in black circles; rat in light and dark green squares; dog in red triangles.

Concentration time course data were dose normalized to allow for cross-comparison between studies given the varied dose levels of administered API (Fig 2). Plasma concentration profiles of total and released API were sampled up to 120 hours post dose, enabling a longitudinal head-to-head comparison across species: dose normalized exposures in plasma were found to be indistinguishable in mouse, rat and dog. Concentration profiles in liver and spleen however were not equally sampled across species. While liver and spleen concentrations were extensively investigated in mouse, measurements from rat and dog were sparse. In rat only one time point was obtained for liver and none for spleen. In dog two time points were obtained: one at time = 1 h–proximal to the time of maximum exposure (Tmax)–and one at the end of study at 120 h (Tlast) for both liver and spleen. Recognizing the incomplete dataset, rat and dog dose normalized concentrations in the liver were in a similar range compared to mouse. Spleen concentrations in dog appeared to follow a somewhat different profile compared to mouse: at t = 1h total and released levels in dog were elevated, whereas at t = 120h dog concentration levels were similar or lower compared to mouse.

### Un-conjugated API clearance rate in mouse, rat and dog

**Fig 3:**
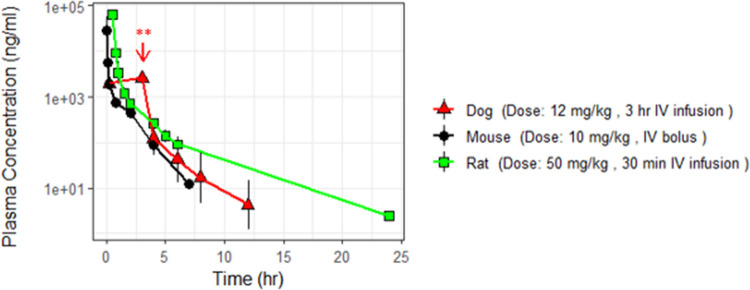
Plasma concentration time course profiles of the un–conjugated API administered across species. The API was administered as an IV bolus in mouse (black circles), as a 30–minute IV infusion in rat (green squares) and as a 3–hour IV infusion in dog (red triangles). ** Red arrow denotes end of 3h infusion in dog.

The un-conjugated API clearance rate (*CL*), assumed equal to the nanoparticle-released API *CL*, was assessed as a model-input parameter likely distinct across species. As part of this work, predicted metabolic API *CL* values, derived from the *in-vitro* intrinsic clearance (Cl_int_) in hepatocyte stability assays, were compared against *in-vivo* systemic *CL* values in mouse, rat and dog. Defining the deviations between predicted and measured API *CL* across species was an integral component of the translational strategy towards simulating clinical profiles, where *in-vivo* data pertaining to the API clearance are absent. Early work in preclinical species showed minimal extrahepatic clearance. Cl_int_ values were therefore scaled using a mathematical model of liver perfusion, known as the well-stirred model [15, 16], to provide predictions of blood *CL* across species (Eqs 1–2). Predicted blood clearance rates were estimated at 2 L/h/Kg, 2.8 L/h/Kg and 2.5 L/h/Kg for mouse, rat and dog respectively (Table 2). We then investigated the in-vivo pharmacokinetic profile of the un-conjugated API administered as an IV bolus of 10 mg/kg in mouse, a 30-minute infusion of 50 mg/kg in rat and a 3-hour infusion of 12 mg/kg in dog as shown in Fig 3. Dose levels of un-conjugated API were chosen to match the doses utilized in nanoparticle biodistribution experiments. Measured *in-vivo* blood CL values, derived by implementing NCA on the IV concentration profiles of the API, were found equal to 2.3 L/h/kg, 2.1 L/h/Kg and 1.5 L/h/kg in mouse, rat and dog respectively, demonstrating less than 2-fold differences from predicted values across all species. Note that, blood clearance constitutes a direct input to the model, via parameter *CL* in Eq 5; released and nanoparticle-conjugated API equations within the PBPK model are defined for the blood compartment (Eqs 4 and 14), with blood API concentrations subsequently converted to plasma concentrations to ensure direct comparison with available PK data (Eqs 9 and 19).

**Table 2:** Predicted and measured blood and plasma clearance rates (*CL*) of API in mouse, rat and dog.

Species	Dose (mg/kg)	Administration	Plasma AUC (μg/ml *h)	Hep Cl_int_ (μl/min/e6 cells)	In-vitro Derived Predicted Blood/Plasma CL (L/h/Kg)	In-vivo Blood/Plasma CL (L/h/kg) (SD)*
Mouse	10	IV bolus	5.6	36	2 / 1.7	2.3 (0.8) / 2 (0.7)
Rat	50	30 min infusion	28	48.7	2.8 /2.4	2.1 (0.1) / 1.8 (0.1)
Dog	12	3 h infusion	8.9	98.3	2.5 / 2.4	(0.4) / 1.5 (0.4)

*SD = standard deviation

### PBPK model: Projections of total and released API from mouse to rat and dog

**Fig 4:**
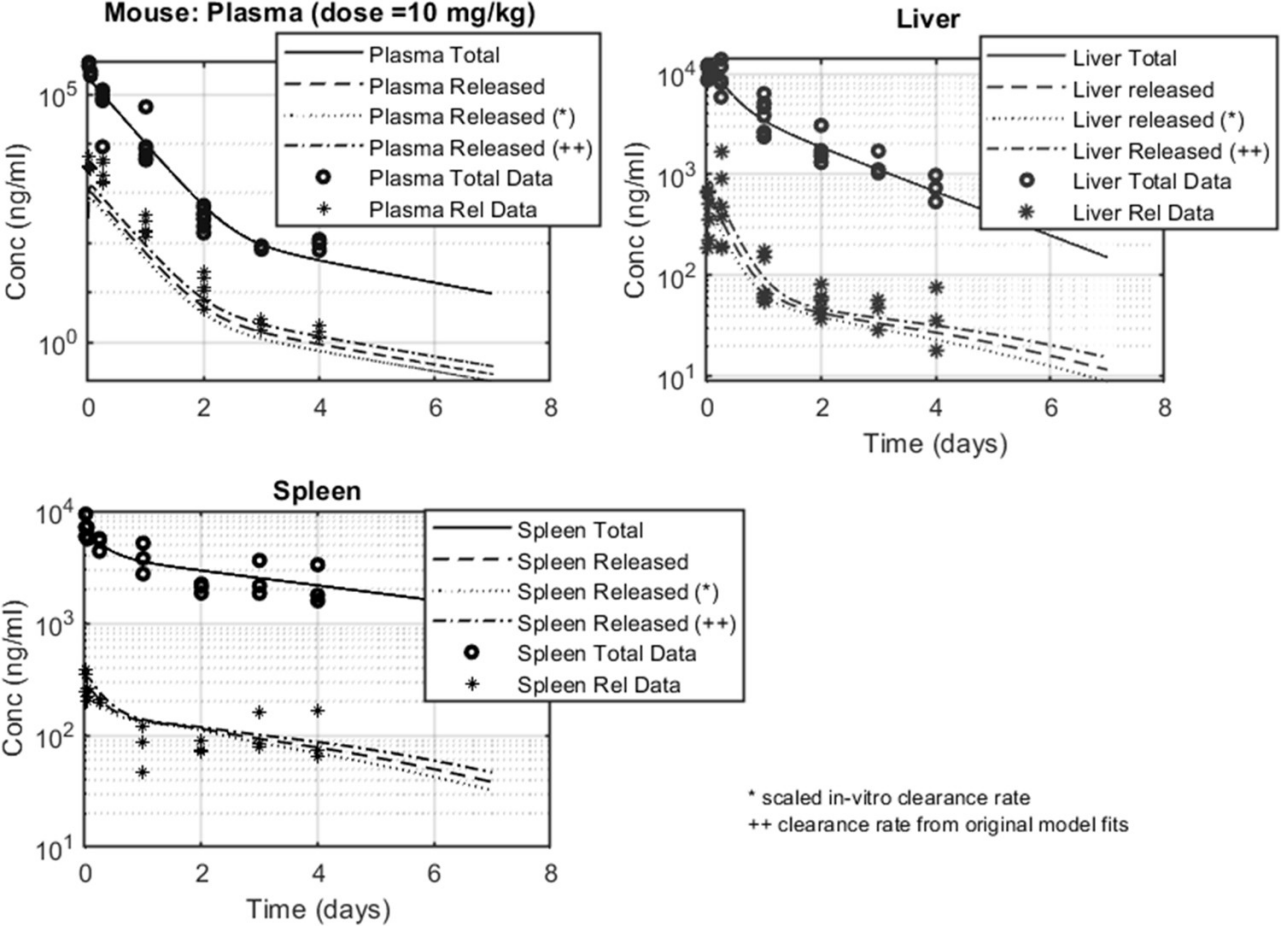
Mouse biodistribution data and model fits in plasma, liver and spleen. Total API data is represented by open circles and released API data by stars. Total API simulated profiles are shown in solid lines; released API simulated profiles are shown in dotted lines (in–vitro scaled API CL value = 2 L/h/Kg), dashed–dotted line (value obtained from previous publication CL = 1 L/h/Kg) or dashed line (in–vivo/measured API CL = 2.3 L/h/Kg).

The main objective of this work was to obtain a quantitative understanding around the biodistribution kinetics of the nanoparticle and its released API across different species. For this purpose, we utilized a PBPK model previously developed for mouse [12] and scaled it to rat and dog. The model as described in the Materials and Methods section was used to project total and released API concentrations in plasma, liver and spleen compartments. Model parameters for the mouse model, summarized in Tables 4 and 5, were derived from our previous work [12]. An additional component in the simulations presented in Fig 4 was the assessment of the API clearance rate (*CL*). The nominal *CL* value, as previously established [12], was 1 L/h/kg. However, as nanoparticle-released and un-conjugated API clearance kinetics were assumed identical, we additionally varied *CL* between 2 L/h/Kg, scaled from the *in-vitro* mouse hepatocyte Cl_int_, and 2.3 L/h/Kg, computed from the NCA of the IV PK profile of the unconjugated API (Table 2). The increase in *CL*, from 1 to 2.3 L/h/Kg, yielded less than two-fold reductions in the simulated released API profiles in plasma, liver and spleen. Total API profiles were 10-100-fold greater compared to released API profiles and therefore remained constant as *CL* was varied.

**Fig 5:**
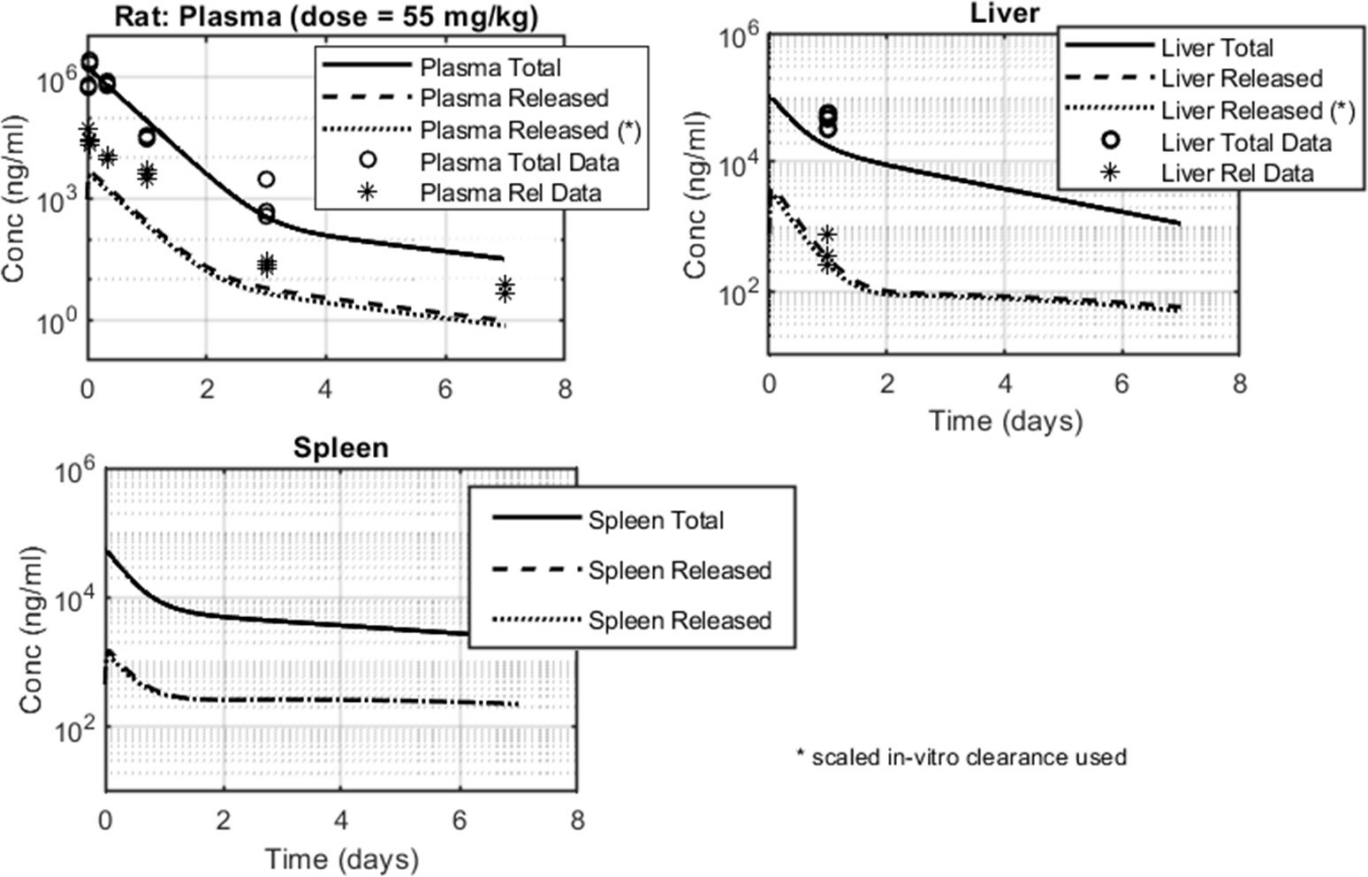
Rat biodistribution data and model fits in plasma, liver and spleen. Total API data is represented by open circles and released API data by stars. Total API simulated profiles are shown in solid lines; released API simulated profiles are shown in dotted lines (in–vitro scaled API CL value = 2.8 L/h/Kg), or dashed line (in–vivo/measured API CL = 2.1 L/h/Kg.

Next steps involved the exploration of the rat biodistribution profile (Fig 5). The PBPK model developed for mouse was scaled to simulate rat profiles; model parameters values are discussed in the Materials and Methods section and are summarized in Tables 4 and 5. The API clearance rate, *CL*, was allowed to vary between 2.8 L/h/kg, as scaled from the *in-vitro* rat hepatocyte Cl_int_, and 2.1 L/h/Kg, as computed from the NCA of the IV PK profile of the unconjugated API (Table 2). As previously mentioned, while plasma PK was extensively sampled, only one time point was obtained from liver and spleen tissues were excluded. Model performance could therefore be assessed mainly from the plasma compartment. Model projections accurately described total API concentrations in plasma, capturing absolute concentration levels as well the shape of the PK profile. With regards to the released API concentrations in plasma, the model recapitulated the observed biphasic profile but underpredicted the data by approximately 3-5-fold. The model performed well for liver: projections were within 2-fold from total API concentration datapoints, while matching released API concertation levels at time = 1 day. A biphasic profile was also projected for total and released API concentrations in spleen.

**Fig 6:**
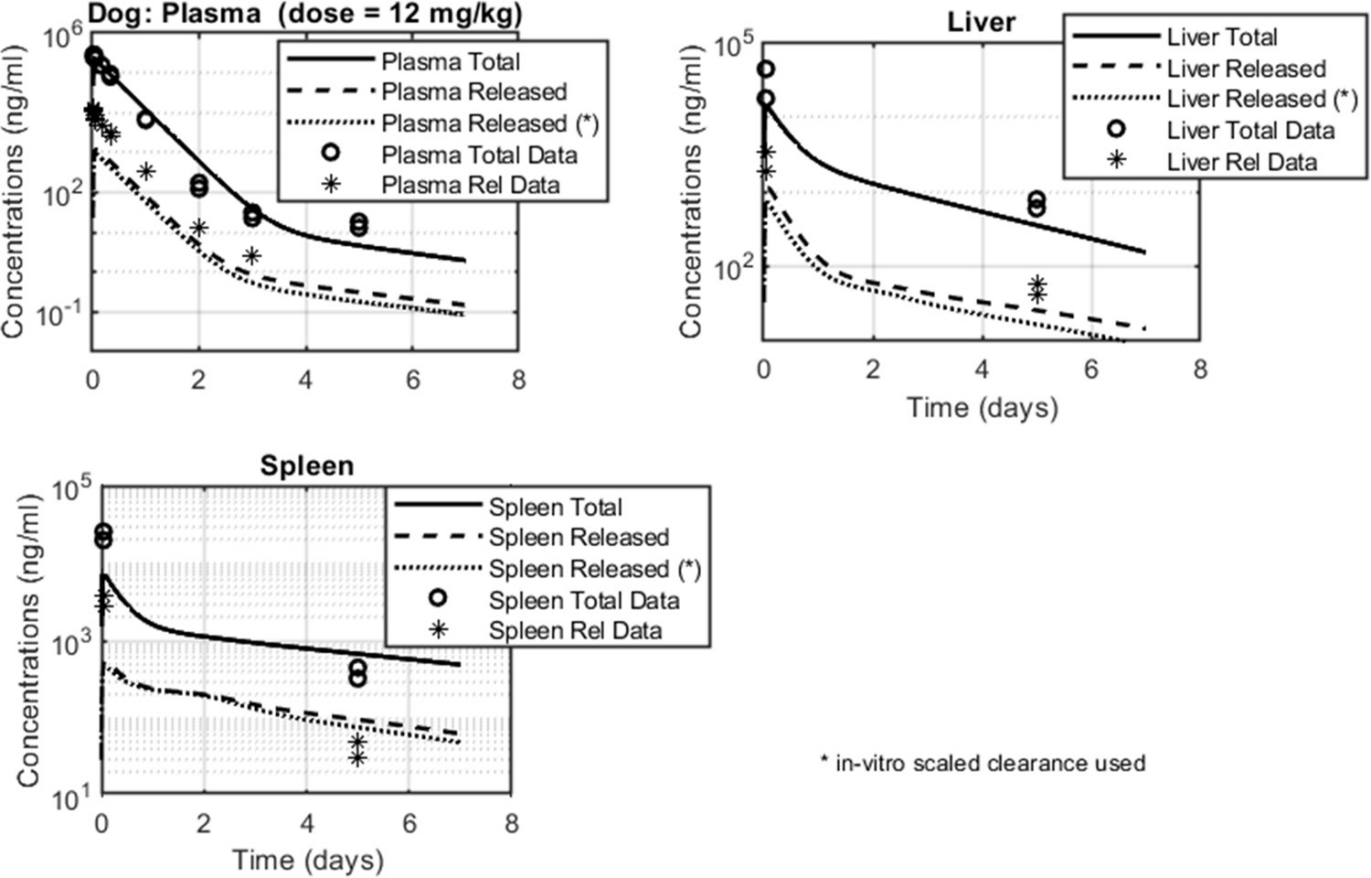
Dog biodistribution data and model fits in plasma, liver and spleen. Total API data is represented by open circles and released API data by stars. Total API simulated profiles are shown in solid lines; released API simulated profiles are shown in dotted lines (in–vitro scaled API CL value = 2.5 L/h/Kg), or dashed line (in–vivo/measured API CL = 1.5 L/h/Kg).

Next, the PBPK model was scaled from mouse to dog, with relevant parameter values summarized in Tables 4 and 5. As before, the API clearance rate (*CL*) was allowed to vary between 2.5 L/h/kg, scaled from the *in-vitro* dog hepatocyte Cl_int_, and 1.5 L/h/Kg, as computed from the NCA of the IV PK profile of the unconjugated API (Table 2, Fig 6). As discussed, plasma PK profiles contained more datapoints compared to tissues. Unlike rat however, liver and spleen datasets included two timepoints: one at Tmax and the other at Tlast. The model was able to accurately describe the plasma PK of total API, matching measured concentration levels and replicating the biphasic profile. Released API levels in plasma however were underpredicted by 3 to 4-fold, similar to the rat. The model performed well when projecting liver total and released API levels: while simulated liver profiles were lower compared to measured values, differences remained within 2-fold. The model-derived PK profile in spleen, despite having a reduced maximum-to-trough concentration ratio than what was experimentally observed, was able to capture total and released concentration data adequately.

**Fig 7:**
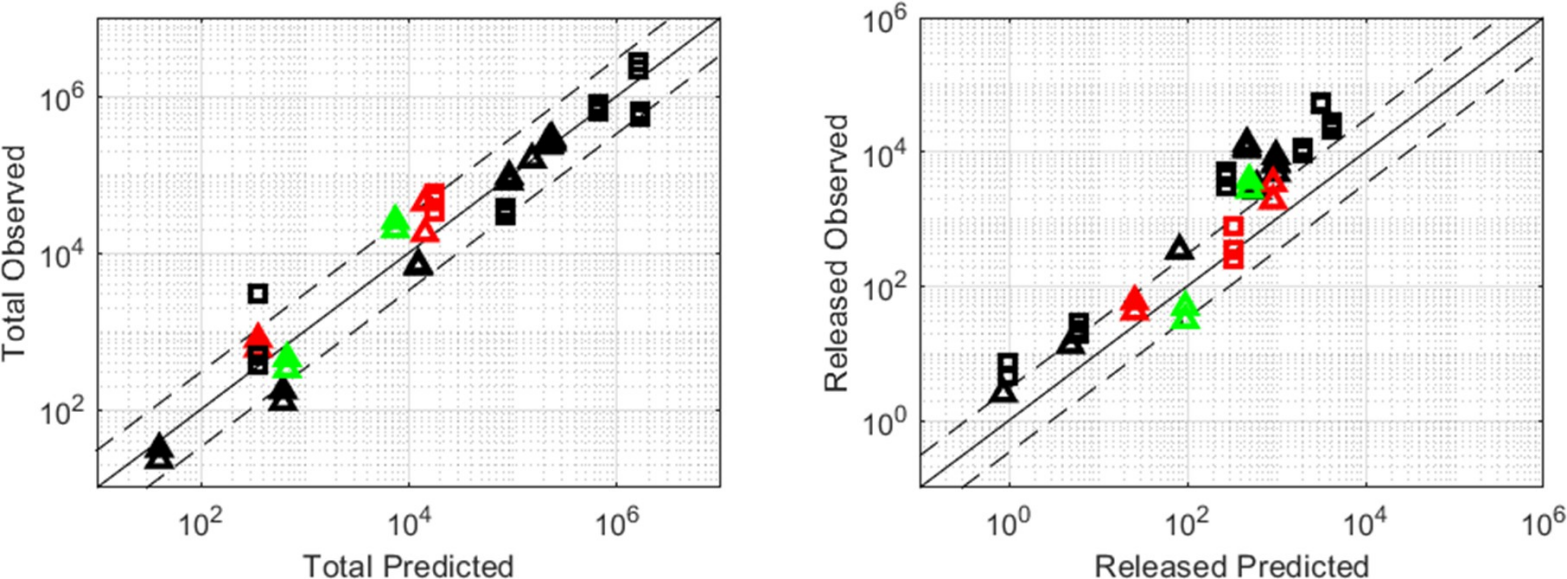
Observed vs predicted total and released concentrations in plasma, liver and spleen across species. Plasma is shown in black; liver in red; spleen in green. Dog is displayed by triangles and rat by squares. Solid black line represents the line of unity; dashed black lines represent 3–fold from unity.

A visual comparison of observed versus predicted concentrations is a typical goodness of fit plot providing an overall summary of the model performance. Simulated values within 3-fold from measured concentrations were considered correctly predicted. Assessments of prediction accuracy within 3-fold multiples are commonly used in drug discovery [17–19]. Total API in plasma, liver and spleen shown in Fig 7 were within 3- fold of unity for both rat and dog. Released API in liver and spleen also fell within the 3-fold specification. By contrast, released concentrations in plasma appeared to deviate up to 5-fold from measured values at earlier timepoints, characterized by higher API concentrations, while falling in line with expected values at later time points. Of note: the *CL* value for these simulations was set to 2.1 L/h/kg and 1.5 L/h/kg for rat and dog respectively, i.e. equal to the unconjugated API clearance rate as measured in-vivo.

### Sensitivity analysis on dog PBPK model

To further investigate the released API dynamics, we conducted a sensitivity analysis on relevant model parameters. While the model performs similarly between species, the dog PBPK model was selected for this analysis given the more complete dataset compared to rat. A five-fold fluctuation was implemented on nominal values of the following parameters: the clearance rate (*CL*), the volume of distribution (*V*_*b*_) and the intercompartmental clearance rate of the released API (*Q*_*BR*_), the apparent peripheral volume or “rest” (*V*_*R*_), the max binding capacity in liver (*B*_*maxL*_), the dissociation constant in liver (*K*_*DL*_) and the non-specific partition coefficient (*P*_*TL*_). Variations in *V*_*b*_, *Q*_*BR*_ and *V*_*R*_ produced minimal changes in PK levels across all compartments and are therefore not shown. Fluctuations in the *B*_*maxL*_, *K*_*DL*_ and P_*L*_ did not affect concentration levels in plasma but did generate changes in the exposure profiles of released API in liver (S1-S3 Figs in S1 File).

**Fig 8:**
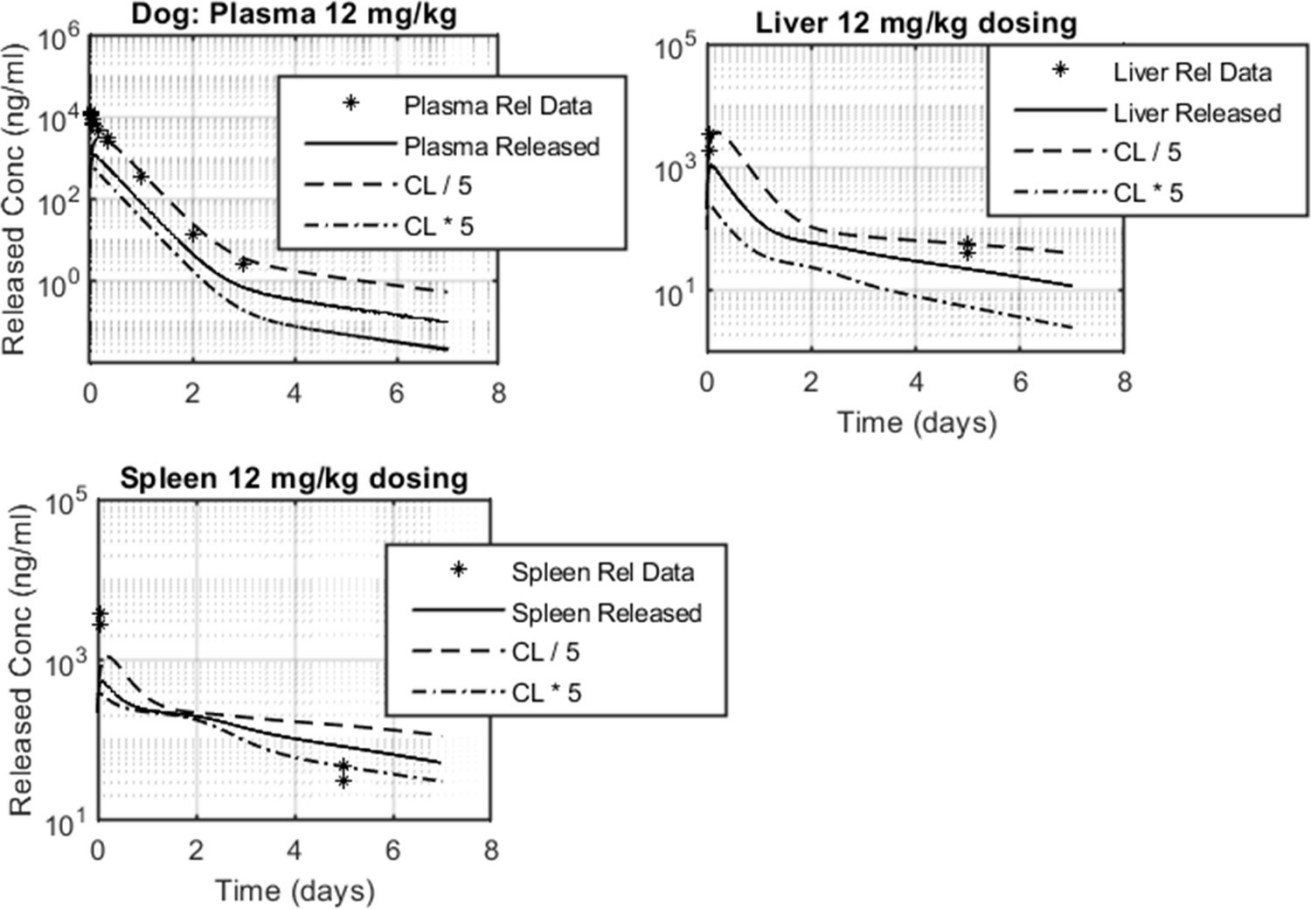
Released API concentration levels in dog as a function of the API clearance rate (CL). Data are represented in asterisks; simulated released API profiles are shown in solid line for nominal CL value, dashed line for 5–fold reduction in CL and dashed–dotted line for 5–fold increase CL.

The released API outputs were found to be most sensitive to the nanoparticle-released API clearance rate, *CL*, which as previously noted was assumed identical to the un-conjugated API clerance. The nominal value of this parameter was set to 1.5 L/h/Kg, as measured *in-vivo* upon administration of a single dose of the unconjugated API. Five-fold changes in the *CL* nominal value visibly shifted released API profiles across plasma, liver and spleen with lower *CL* values generating improved fits to available data (Fig 8). As previously discussed, the projected maximum-to-trough concentration ratio in spleen was lower than what was experimentally observed. Fluctuating *CL* values preserved the biphasic profile in spleen shifting the absolute concentration levels. As before, total API levels across tissues were 10–100-fold greater compared to released API concentrations and therefore remained constant when imposing fluctuations across the selected model parameters.

### Simulated human total and released API profiles

**Fig 9:**
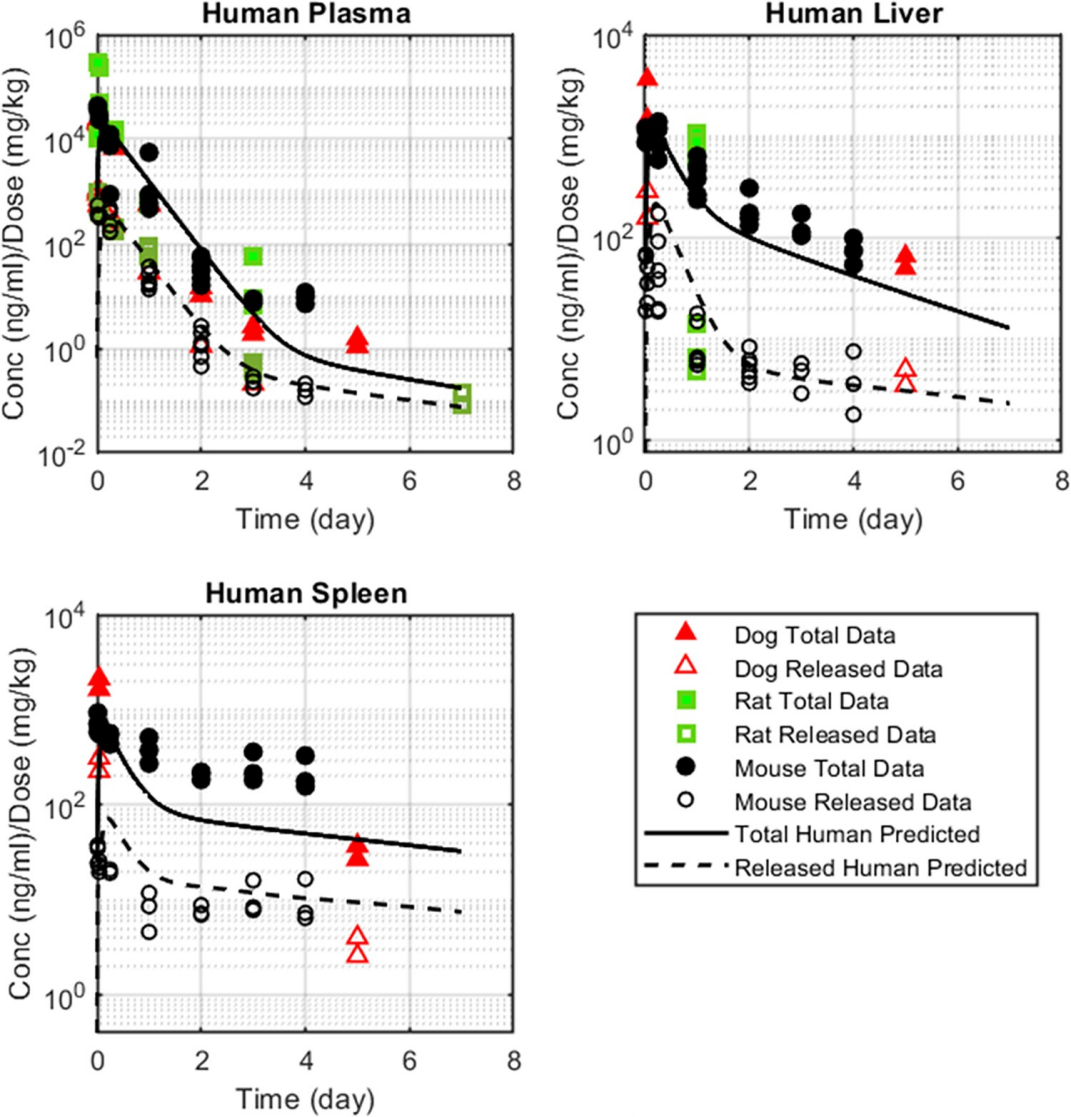
Human dose–normalized simulations plotted against preclinical dose–normalized concentration data measured across species. Dog is shown in red triangles; rat in green squares; mouse in black circles. Total concentrations are represented by closed symbols; released concentrations by open symbols.

Total and released API concentration profiles were projected for human, further comparing simulation outputs against available preclinical data. Model parameters for human were derived similarly to rat and dog: allometry was applied to scale the API central and peripheral compartment volumes, the API intercompartmental clearance, partition coefficients and extravasation rates of the nanoparticle-conjugated API, as shown in Eqs 22–25. All parameter values are summarized in Tables 4 and 5. On the basis of the previously established interspecies in-vitro/in-vivo correlation of the API clearance (Table 2), the released API human *CL* was set to 0.32 L/h/Kg, as derived from the scaling of the in-vitro human hepatocyte Cl_int_ of 15.5 μl/min*e6 cells (Eqs 1–2). Simulations were carried out for a 10 mg/kg dose; model-derived concentration profiles shown in Fig 9 are dose normalized to allow for a direct cross-comparison with mouse, rat and dog. Simulated dose-normalized concentration profiles for human are shown to align well with total and released concentration data in plasma, liver and spleen as measured from preclinical species.

## Discussion

Within this manuscript, comparable dose-normalized concentration profiles of total and released API in plasma and tissues across species have been demonstrated. This finding is in agreement with reported liposomal and polymeric nanoparticle interspecies PK studies; while noting that the majority of published reports have measured total API or nanocarrier concentrations [6, 13, 14, 20], Eliasof et al, investigating the biodistribution of CLRX101 –a clinically tested polymeric nanoparticle covalently conjugated to camptothecin (CPT)–successfully measured released API exposures demonstrating dose proportionality across species [21]. Plasma dose normalized AUCs appeared within two-fold in mouse, rat and dog, in turn indicating comparable clearance rates across tested species. Measured half-lives of total API in plasma across species and doses were nearly identical, ranging between 6.8–15 h, interestingly matching the time required for the release of 50% of the conjugated API (referred to as the *T50*), previously estimated at 5.5 h [12]. These observations align with our model structure, which has assumed that systemic elimination of total API is driven mainly by the release of the API from the nanoparticles, thereby omitting a separate nanoparticle clearance term. The apparent clearance rates of the released API were also demonstrated comparable across species, however, were significantly lower compared to the clearance rates derived from the un-conjugated API. Hence, dose normalized AUCs of the nanoparticle-released API were 6-12-fold higher compared to dose normalized AUCs of the unconjugated API in conventional formulation across all species (% bioavailability ranged ~600–1200% as presented in S1 Table in S1 File). In our prior study we had attributed the differences in dose normalized AUCs across formulations partly to the uncertainty around the estimated Co (maximal concentration upon IV bolus administration) given the sharp biphasic profile of the unconjugated API. However, in rat and dog PK studies the unconjugated API was dosed as a 30-minute IV infusion eliminating this concern. Similar deviations between measured and expected released API concentration profiles have been previously reported in the literature across different modalities, nanoparticles as well as antibody drug conjugates [21, 22]. Observed differences were mainly attributed to uncertainties in protein binding kinetics of the API in plasma, with correction factors subsequently applied to accurately fit and mathematically describe measured concentrations, highlighting the possibility of parameter misspecifications when quantifications of API pharmacokinetics are transferred across formulation types and modalities.

The ability to measure both total and released API concentrations in plasma and tissues has enabled the evaluation of underlying dynamics that may otherwise be missed. An important observation from our previous work was the range of release rate constants across compartments, where 50% of the API was released at 5.5h, 43h and 110h in plasma, liver and spleen respectively. The dendrimer-drug covalent bond was shown to be pH sensitive *in-vitro*, with slower release rates noted for more acidic conditions [12]. Such dependencies have been shown for other nanoparticles in the literature [10], with CLRX101, covalently conjugated to camptothecin (CPT) as a notable example [21, 23]. We had previously rationalized the range of observed release rates across tissues as part of the compartmentalization of nanoparticles in distinct tissue subsections, such as the lysosome of Kupffer cells comprising the RES, which may be characterized by varied pH levels. When scaling the PBPK model, release rate constants across plasma, liver and spleen were assumed identical across mouse, rat, dog and human. The agreement of projected and measured interspecies concentrations provides confirmation on the assumption around the conservation of API release kinetics.

Our main objective was to evaluate the utility of a previously developed PBPK model parameterized for mouse to prospectively capture concentration time course data in higher order species. Simulated values for total API concentrations across compartments as well as released API in liver and spleen were within 3-fold from measured concentration data and were therefore considered as accurately predicted. Released API in plasma however was underpredicted, in particular at the earlier timepoints, across all preclinical species. These discrepancies are likely due to a parameter misspecification; therefore, as a next step we conducted a sensitivity analysis on relevant model parameters investigating the underlying dynamics leading to the deviation of simulated released API concentrations from measured data in plasma. A subset of parameters–such as the max binding capacity in liver (*B*_*maxL*_), the dissociation constant in liver (*K*_*DL*_) and the non-specific partition coefficient (*P*_*TL*_)–were shown to affect simulated released API profiles in liver but not in plasma or spleen. As previously discussed, released API uptake in liver follows non-linear kinetics, consisting of two components: 1) non-specific binding, captured by parameter *P*_*L*_ and 2) saturable kinetics, captured by *B*_*maxL*_ and *K*_*DL*_, defined as the maximum binding capacity and the dissociation constant of the released API at the binding site, respectively [12]. Fluctuations in *P*_*L*_ affected early timepoints whereas fluctuations in *B*_*maxL*_ and *K*_*DL*_ affected later timepoints of the simulated released API PK profiles in liver. Plasma profiles remained unchanged throughout these simulations despite the released API being cleared in its entirety from the liver compartment, thereby excluding the extent of released API accumulation in liver as the rate limiting step shaping the systemic API profile. Furthermore, projected concentration profiles were shown to be most sensitive to fluctuations in the API clearance rate (*CL*). While the API *CL* demonstrated a strong in-vitro/in-vivo correlation (IVIVC within 2-fold) when either scaled from *in-vitro* hepatocyte Cl_ints_ or computed from the IV PK profile of the unconjugated API, nominal *CL* values required a 5-fold reduction to achieve improved fits of released plasma API. We hypothesize the differential distribution of the API within liver sub compartments, when administered in its unconjugated versus nanoparticle format, ultimately affecting its metabolic rate, as the contributor to the disparate clearance outcomes. This work has highlighted the importance of properly evaluating the apparent *CL* of the API, conducting a sensitivity analysis around this parameter as part of exploratory work and prospective simulations.

Dynamics not incorporated explicitly in the model involved the immunological contribution and potential species differences in the macrophage activity, influencing nanoparticle endocytosis and clearance [24]. Modeling work by Lin et al on polyethylene glycol (PEG) gold nanoparticles included mathematical representation of the endocytosis by phagocytic cells, which were considered separately in liver, spleen, kidneys and lung, accounting for the maximum uptake capacity of each organ. Endocytic parameters were estimated iteratively for each studied species and for low versus medium doses [25]. Other PBPK models have similarly considered macrophages as a separate sub-compartment within each organ [14]. In this context, our model differs from published studies: nanoparticle extravasation rates employed within our work have lumped individual processes such as the transcytosis, phagocytosis and the potential recycling of nanoparticles back to circulation owing to the lymphatic system. Extravasation rates were scaled from mouse utilizing blood flow rates in tissues as the scaling factor between species. Human extravasation rates in liver and spleen were therefore estimated approximately 10-fold slower compared to mouse, aligning well with published studies also reporting lower human-derived parameter values descriptive of nanoparticle kinetics compared to mouse or rat [25, 26].

While this work introduces a unified computational framework describing the biodistribution of dendritic nanoparticles across species, there are a few limitations to be noted. The heterogeneity in experimental design and time point selection across species in liver and spleen tissues do not allow for a rigorous assessment of the model performance longitudinally in these tissues. Rather the model accuracy was assessed by looking at the totality of the data and acknowledging that it successfully captures the measured concentrations of released and total API across compartments. The implementation of the PBPK model also enables the visualization of full concentration time course profiles across the selected timeframe, which is informative when evaluating the exposure trends beyond the measured data points that are often limited in tissues. The established model performance as well as the reliable dose-proportional pharmacokinetic data across species has enabled clinical projections. While theoretical, human concentration time course profiles are comparable to dose normalized preclinical data aligning well with observations across mouse, rat and dog. As a note, applying the same analysis to polymeric or other nanoparticle types requires careful consideration. Serum protein adhering to the surface of nanoparticles form the “protein corona” that ultimately affects the rate of cellular uptake and systemic elimination. Quantifying the interactions between nanoparticles and the RES and defining this relationship is integral in understanding potential deviations from expected nanoparticle exposure profiles [27–30]. Despite such differences, the work presented within this manuscript provides a mathematical framework enabling the projections of concentrations across species while offering the potential to include additional mechanistic components specific to the nanoparticle studied.

## Materials and methods

### Predicted metabolic clearance by hepatocyte Clint scaling

Methods for hepatocyte Cl_int_ measurements, utilized within this manuscript, have been described in detail elsewhere [17]. In brief, hepatic Cl_int_ values were scaled based on Eq 1:

$${CL}_{scaled,u}=\frac{Clint}{fuinc}*hepatocellularity\;(x\;10^{6})/g\;liver*\frac{liver\;weight\;(g)}{body\;weight\;(kg)}\;$$ (1)

Fuinc is the incubational protein binding, equal to 0.007. Other parameter values across species are listed in Table 3.

**Table 3:** Summary of physiological parameters used for scaling the hepatocyte Cl_int_ across species.

Species	Hepatocellularity (x10^6^) / g liver	Liver weight (g)	Body weight (Kg)
Mouse	125	1.25	0.02
Rat	163	10	0.25
Dog	169	384	10
Human	120	1680	70

The scaled unbound intrinsic metabolic CL (CL_scaled,u_) was then incorporated to the well stirred model (Eq 2), also applying a regression offset of 3 to correct for the systematic underprediction of in vivo CL as discussed in previous analyses [17]:

$${Cl}_{\;met}=\frac{Q_{\;h}\;*fu,b*{CL}_{scaled,u}}{Q_{\;h\;}+fu,b*{CL}_{scaled,u}\;}\;$$ (2),

where *Q*_*h*_ is the hepatic blood flow (ml/min/kg), *fu*,*b* is the free fraction in blood.

$${fu}_{,blood}=\left[\frac{fup}{BPR}\right]$$ (3)

,where *fup* is the protein binding of the API in plasma and *BPR* the blood-to-plasma partitioning ratio of the API.

### In-vivo studies in mouse, rat and dog

In vivo PK studies with the nanoparticle used four male beagle dogs which were sourced from a colony held at Charles River Laboratories and animals were group housed throughout the study apart from during dosing and feeding. Each animal received a single intravenous infusion of the nanoparticle over 30 minutes at a target dose level of 24 mg/kg/hour (12 mg/kg). This dose level was considered to be the NOEL (No Observed Effect Level). Formulations were prepared at a concentration of 13.2 mg/mL and administered at a dose volume of 5 mL/kg. Blood samples were collected from 2 dogs pre-dose, at the end of infusion, and 1 hour post dose. In a further two dogs, blood samples were collected pre-dose, at the end of infusion, 1, 4, 8, 24, 48, 72 and 120 hours post dose. At necropsy, samples of pancreas, liver, spleen and muscle were taken for tissue analysis and mass spec imaging. Animals were humanely terminated according to local SOPs for tissue sampling after collection of the final blood samples at either 1 hour or 120 hour post dose.

Dogs assigned to the study with the API were sourced from Envigo RMS (UK) Limited, Shaw’s Farm, Blackthorn, Bicester, Oxon, UK. Animals were housed in groups of 2 or 3 by sex in custom designed dog pens with an area of at least 2.25m^2^ for each dog. Animals were separated for dosing, post dose observations and feeding. All animals received a three hour intravenous infusion once a week on 5 occasions at doses of 0, 2, 6 or 12/20 (M/F) mg/kg (according to group allocation) at a dose volume of 10 mL/kg. PK sampling was on Days 1 and 29. A 0.5 mL blood sample was taken at 15 minutes, 3 hours (immediately after the end of infusion) and then 4 hours, 6 hours, 8 hours and 12 hours from the start of infusion. At the end of the experiment, animals were humanely terminated according to local SOPs for tissue sampling.

Both dog studies were run in the UK. All in vivo animal studies underwent ethical review by the CRL Edinburgh AWERB (Animal Welfare and Ethical Review Body), under the appropriate (PPL) Project License that is reviewed and approved by the UK Home Office; and follow the principles of the 3Rs. Each study is carefully considered and justified to ensure that: the study is scientifically necessary; there is no reasonably practicable alternative to the use of animals in part or all of the study (Replacement); the study is designed and analyzed to be robust and reproducible in achieving its scientific objective (Reduction) and the study is designed to exploit the latest scientific knowledge and in vivo technologies in order to minimize pain and distress to the animals involved (Refinement). The UK Home Office controls scientific procedures on animals in the UK and does so by the issue of licenses under the Animals (Scientific Procedures) Act 1986. The regulations conform to the European Convention for the Protection of Vertebrate Animals Used for Experimental and Other Scientific Purposes (Strasbourg, Council of Europe) and achieve the standard of care required by the US Department of Health and Human Services’ Guide for the Care and Use of Laboratory Animals. The Home Office license governing this study strictly specifies the limits of severity of effects on the animals. Veterinary care was available throughout the course of the study and animals were examined by the veterinary staff as warranted by clinical signs or other changes. All veterinary examinations and recommended therapeutic treatments, if any, were documented in the study records. In the event that animals showed signs of illness or distress, the responsible veterinarian may make initial recommendations about treatment of the animal(s) and/or alteration of study procedures, to address a potentially life-threatening situation, or to alleviate acute severe pain. Treatment of the animal(s) for minor injuries or ailments may be approved without prior consultation with the Sponsor representative when such treatment does not impact fulfilment of the study objectives. If the condition of the animal(s) is such that emergency measures must be taken, the Study Director and/or veterinarian has authority to act immediately at his/her discretion to alleviate suffering. From the available information, the procedures described in the protocols were not anticipated to cause any effects which exceed the severity limit of the procedure. Any animal which showed unacceptable reactions would have been euthanized or other actions taken as required by the Home Office to alleviate distress. However, this was not required on these studies. Animals were euthanized at the end of study by intravenous injection of sodium pentobarbital, followed by exsanguination.

In vivo mouse studies with the nanoparticle used twenty-one SCID CB-17 mice sourced from Charles River Laboratories. Mice received a single dose of the nanoparticle via an intravenous bolus injection at dose levels of 10 mg/kg (dose volume of 5 ml/kg). Blood samples were collected from 3 mice at each of the selected timepoints of 20 minutes, 1h, 6 h, 24 h, 48 h, 72 h and 96 hr post nanoparticle dosing. In vivo mouse studies with the API used three female SCID CB-17 mice sourced from Charles River Laboratories. Mice received a single dose of the API via an intravenous bolus injection at a dose level of 10 mg/kg (formulation concentration of 2 mg/mL and dose volume of 5ml/Kg). Blood samples were collected 2, 5, 15 and 45 minutes post dose, as well as 2, 4, 7 and 24 hours post dose of the API administration. Animals from both mouse studies were housed in individually ventilated cages (IVCs; Tecniplast, Italy) at a temperature of 68°F +/-3°F, humidity of 45% to 70%, 60–70 air exchanges per hour in the cages, and a 12/12-hour light/dark cycle with the lights on at 6:00 AM. The maximum caging density was five mice of the same sex. Animals were humanely euthanized by carbon dioxide asphyxiation performed using gas regulators following collection of the final blood sample and select tissues were collected at necropsy. Animal health and well-being were observed frequently over the course of studies and assessed at the time of test article administration(s) and all collection time points during each experiment. Criteria used to assess animals includes body weight, body condition score, general appearance and behavior. Nutritional supportive care (NutraGel, Bio-Serv, Flemington, NJ, USA) and supplemental hydration (Pure Water Gel, Bio-Serv, Flemington, NJ, USA), when required, were provided under the direction of the Attending Veterinarian. All procedures were reviewed and approved by the Institutional Animal Care and Use Committee, AstraZeneca R&D Boston and conducted under an approved IACUC protocol in compliance with the Guide for the Care and Use of Laboratory Animals, 8^th^ Edition (National Research Council, National Academies Press, Washington D.C., USA).

In vivo rat studies with the nanoparticle used nine Male Han Wistar rats sourced from Charles River Laboratories. All animals received a 30-minute intravenous infusion of the nanoparticle at day1 and day 8 at dose levels of 55, 110 or 505 mg/kg (dose volume of 10 ml/kg). Blood samples were collected 0.5, 1,8, 24 and 72 hr post dose at day 1. Tissue samples were collected at 24hr post dose at day 8. In vivo rat studies with the API used two male Han Wistar rats sourced from Charles River Laboratories. Rats received a single dose of the API via a 30-minute intravenous infusion at a dose level of 50 mg/kg (formulation concentration of 10 mg/mL and dose volume of 5 ml/kg). Blood samples were collected 30, 45, 60, 90 minutes post dose, as well as 2, 4, 5, 6 and 24 hours post dose. The animals were housed two per cage, while during the tail vein infusion they were housed in individual cages and returned to pair housing at the completion of the dosing period. Animals were housed in individually ventilated cages (IVCs; Tecniplast, Italy) at a temperature of 68°F +/-3°F, humidity of 45% to 70%, 60–70 air exchanges per hour in the cages, and a 12/12-hour light/dark cycle with the lights on at 6:00 AM. Animals were humanely euthanized by carbon dioxide asphyxiation performed using gas regulators. Animal health and well-being were observed frequently over the course of studies and assessed at the time of test article administration(s) and all collection time points during each experiment. Criteria used to assess animals includes body weight, body condition score, general appearance and behavior. Nutritional supportive care (NutraGel, Bio-Serv, Flemington, NJ, USA) and supplemental hydration (Pure Water Gel, Bio-Serv, Flemington, NJ, USA), when required, were provided under the direction of the Attending Veterinarian. All procedures were reviewed and approved by the Institutional Animal Care and Use Committee, AstraZeneca R&D Boston and conducted under an approved IACUC protocol in compliance with the Guide for the Care and Use of Laboratory Animals, 8^th^ Edition (National Research Council, National Academies Press, Washington D.C., USA).

Noncompartmental analysis (NCA) on collected plasma concentration profiles was implemented using Phoenix32

#### Treatments

The nanoparticle was formulated in 10 mM acetate buffer 175 pH 5.0 with 5% glucose, and the API was formulated in 0.15 M meglumine /30% 176 HP-β-CD and dosed as an intravenous (IV) administration at a volume of 5 mL/kg at the indicated doses.

### Bioanalysis

Detailed descriptions of bioanalytical methods, applied within this work, are found elsewhere [12]. In brief, to assess in-vivo plasma and tissue concentrations, all samples were collected and split in two sets designated for measuring either released API concentrations or total API concentrations. All concentrations were measured using a protein precipitation/liquid-liquid extraction procedure followed by liquid chromatography with tandem mass spectrometric detection (LC-MS/MS). Sample sets, designated for the measurement of released API, were immediately acidified upon collection to halt any further release of the API from the nanoparticles by adding 0.2M citrated commercial mouse plasma in 1:1 (volume: volume) ratio; they were subsequently flash frozen. Sample sets, designated for the measurement of total concentrations did not require the addition of stabilizing agents, however, were immediately flash frozen upon collection. Tissue samples, split into two sets, were homogenized with blank mouse plasma in 1:6 (weight: volume) ratio. This methodology is similar to the one developed for AZD0466 [11], where reduced temperature and pH levels were made use to stabilize nanoparticles and prevent further API release post sample collection.

### Model description

A previously developed physiologically based pharmacokinetic (PBPK) model was utilized to capture the nanoparticle biodistribution dynamics. Detailed description of the model is provided elsewhere [12]. The model consists of two main components describing a) the nanoparticle-released API and b) the nanoparticle-conjugated API dynamics. Model compartments included the blood, liver, spleen and the rest, the latter used for mass balance purposes. The data was plotted using ggplot in R 4.1.1. Matlab R2020b (Mathworks, Natick MA), implementing ode23 to solve the system of ordinary differential equations (ODEs), was used to simulate PK in plasma and tissues. Files containing relevant code can be found in S2 and S3 Files.

#### Released-API equations

Released API amounts in the blood (*A*_*b*_), liver (*A*_*L*_), spleen (*A*_*S*_) and rest (*A*_*R*_) were described using the following equations:

$$\frac{dA_{\;b}}{dt}=-Q_{BL,i\;}\frac{A_{b}}{V_{b}}+Q_{BL,o}\;\frac{A_{L\;}}{V_{L}*K_{BL}}-Q_{BS\;}\frac{A_{b}}{V_{b}}-Q_{BR\;}(\frac{A_{b}}{V_{b}}-\frac{A_{R}}{V_{R}})+{krel}_{b}*X_{b}$$ (4)

$$\frac{dA_{\;L}}{dt}=Q_{BL,i\;}\frac{A_{b}}{V_{b}}-Q_{BL,o}\;\frac{A_{L\;}}{V_{L}*K_{BL}}-CL\frac{A_{L\;}}{V_{L}*K_{BL}}+Q_{BS\;}\frac{A_{s\;}}{V_{S}*K_{BS}}+{krel}_{L}*X_{L}$$ (5)

$$\frac{dA_{\;S}}{dt}=Q_{BS\;}(\frac{A_{b}}{V_{b}}-\frac{A_{s\;}}{V_{S}*\;K_{BS}})+{krel}_{S}*X_{S}\;$$ (6)

$$\frac{dA_{\;R}}{dt}=Q_{BR\;}\left(\frac{A_{b}}{V_{b}}-\frac{A_{r\;}}{V_{R}}\right)+{krel}_{R}*X_{R}$$ (7)

*V*_*x*_ represents the volume of each compartment, *Q*_*BX*_ the intercompartmental blood flow rate and *K*_*BX*_ the tissue/blood partition coefficient; *krel*_*x*_ is the release rate constant and *X*_*x*_ the nanoparticle- conjugated API from respective compartments. Subscript *‘x’* can be substituted by *L*, *S* and *R*, corresponding to the liver, spleen and rest respectively. *CL* represents the clearance of the API from the liver.

To obtain concentration values across compartments, released API amounts were divided by the volume of their respective compartments.


$$C_{blood,released}={\frac{A_{b}}{V_{b}}}_{\;}$$ (8)

$$C_{plasma,released}={\frac{C_{blood,released}}{BPR}}_{\;}$$ (9)

$$C_{liver,released}=\frac{A_{L\;}}{V_{L}}$$ (10)

$$C_{spleen,released}=\frac{A_{s\;}}{V_{S}}$$ (11)

As previously described API uptake in liver and spleen tissues follows non-linear, saturable dynamics. Similar kinetics have been reported throughout the literature [31–34].


$$K_{BL}=\frac{{Bmax}_{L}\;}{C_{blood,released}+{KD}_{L}}+P_{L}$$ (12)

$$K_{BS}=\frac{{Bmax}_{S}\;}{C_{blood,released}+{KD}_{S}}+P_{S}$$ (13)

Where *Bmax*_*L*_, *Bmax*_*s*_ is the maximum capacity of the high affinity binding site; *KD*_*L*_, *KD*_*S*_ the dissociation constants and *P*_*L*_, *P*_*s*_ represent the contribution from non-specific binding in liver and spleen, respectively.

#### Nanoparticle- conjugated API equations

The nanoparticle- conjugated API amounts in the blood (*X*_*b*_), liver (*X*_*L*_), spleen (*X*_*S*_) and rest (*X*_*R*_) were described using the following equations:

$$\frac{dX_{b}}{dt}=-N_{BL\;}\left(\frac{X_{b}}{V_{Nb}}-\frac{X_{L}}{V_{L}*K_{NBL}}\right)-N_{BS\;}\left(\frac{X_{b}}{V_{Nb}}-\frac{X_{s}}{VS*K_{NBS}}\right)-N_{BR\;}\left(\frac{X_{b}}{V_{Nb}}-\frac{X_{R}}{V_{NR}}\right)-{krel}_{b}*X_{b}$$ (14)

$$\frac{dX_{L}}{dt}=N_{BL\;}\left(\frac{X_{b}}{V_{Nb}}-\frac{X_{L}}{V_{L}*K_{NBL}}\right)-{krel}_{L}*X_{L}$$ (15)

$$\frac{dX_{S}}{dt}=N_{BS\;}\left(\frac{X_{b}}{V_{Nb}}-\frac{X_{s}}{V_{S}*K_{NBS}}\right)-{krel}_{S}*X_{S}\;$$ (16)

$$\frac{dX_{R}}{dt}=N_{BR\;}\left(\frac{X_{b}}{V_{Nb}}-\frac{X_{R}}{V_{NR}}\right)-{krel}_{R}*X_{R}$$ (17)

*V*_*Nb*_ and *V*_*NR*_ is the volume of the blood and rest compartments, respectively. The liver and spleen compartmental volumes, *V*_*L*_ and *V*_*S*_, were fixed to their physiological values and were considered identical between the released- and nanoparticle-conjugated API models (Tables 4 and 5). *N*_*BX*_ is the intercompartmental extravasation rate and *K*_*NBX*_ the tissue/blood partition coefficient. The subscript *‘x’* can be substituted by *L*, *S* and *R*, corresponding to the liver, spleen and rest respectively. A separate nanoparticle clearance term was omitted, assuming that systemic elimination of nanoparticles occurs at much longer timescales compared to tissue distribution and in particular API release, with 50% of the API released in plasma by 5.5 hours [12].

**Table 4:** Nanoparticle–released API parameter values.

Parameter (units)	Description	Mouse	Rat	Dog	Human	Notes
BW (kg)	Body Weight	0.02	0.25	10	70	
fup	Plasma Protein binding %	0.03	0.06	0.06	0.03	
BPR	Blood to plasma ratio	0.84	0.84	0.97	0.77	Measured for small molecule
V_b_ (L/Kg)	Volume of distribution in blood; apparent volume of central compartment	0.34	0.67	0.59	0.37	Fit for mouse [12]; scale for other species Eq 22
V_L_ (L/kg)	Liver volume	0.065	0.078	0.048	0.024	Fixed: physiological value *
V_S_ (L/kg)	Spleen volume	0.005	0.0052	0.0036	0.0027	Fixed: physiological value *
V_R_ (L/kg)	Rest volume; apparent volume of peripheral compartment	0.1	0.2	0.19	0.12	Fit for mouse [12]; scale for other species Eq 22
Q_BL,i_ (L/kg/h)	Blood to liver flow rate	8.36	5.25	3.15	1.23	QBL,i—Q_BS_
Q_BL,o_ (L/kg/h)	Liver to blood flow rate	9.1	5.4	3.3	1.3	Fixed: physiological value *
Q_BS_ (L/kg/h)	Blood/spleen flow rate	0.74	0.15	0.15	0.066	Fixed: physiological value *
Q_BR_ (L/kg/h)	Blood/rest flow rate	0.015	0.007	0.0024	0.0013	Fit for mouse [12]; scale for other species Eq 23
CL (L/kg/h)	Released API intrinsic clearance rate	1–2.3	2.1–2.8	1.5–2.5	0.32	Discussed in table 2
Bmax_L_ (ng/ml)	Max binding capacity in liver	49.6	99	85	54	Fit for mouse [12]; scale for other species Eq 24
KD_L_ (ng/ml)	Dissociation constant in liver	0.6	= mouse	= mouse	= mouse	
P_L_	Non-specific binding in liver	0.76	1.5	1.3	0.83	Fit for mouse [12]; scale for other species Eq 24
Bmax_S_ (ng/ml)	Max binding capacity in spleen	125.9	251.8	217.8	138	Fit for mouse [12]; scale for other species Eq 24
KD_S_ (ng/ml)	Dissociation constant in spleen	0.63	= mouse	= mouse	= mouse	
P_S_	Non-specific binding in spleen	0.17	0.33	0.3	0.19	Fit for mouse [12]; scale for other species Eq 24

*Davies et al [5]

**Table 5:** Nanoparticle–conjugated API parameter values.

Parameter (units)	Description	Mouse Value	Rat Value	Dog Value	Human	Notes
V_Nb_ (L/kg)	Volume of distribution in blood, conjugated API	0.085	0.054	0.09	0.074	Fixed: physiological value of blood *
V_NR_ (L/kg)	Rest volume, conjugated API	1-∑(*Vtissues*)	1-∑(*Vtissues*)	1-∑(*Vtissues*)	1-∑(*Vtissues*)	
N_BL_ (L/kg/h)	Blood/liver extravasation rate	0.0003	0.0002	0.0001	0.00004	Fit for mouse [12]; scale for other species Eq 25
N_BS_ (L/kg/h)	Blood/spleen extravasation rate	2e-5	4e-6	4e-6	1.7e-6	Fit for mouse [12]; scale for other species Eq 25
N_BR_(L/kg/h)	Blood/rest distribution rate	0 ^+^	0 ^+^	0 ^+^	0 ^+^	
K_NBL_	Liver/blood partition coefficient	0.9	1.8	1.6	1	Fit for mouse [12]; scale for other species Eq 24
K_NBS_	Spleen/blood partition coefficient	1000	1000	1000	1000	Fixed ^++^
krel_b_ (1/h)	API release rate in blood	0.125	= mouse	= mouse	= mouse	
krel_L_ (1/h)	API release rate in liver	0.016	= mouse	= mouse	= mouse	
krel_S_ (1/h)	API release rate in spleen	0.0063	= mouse	= mouse	= mouse	
krel_R_(1/h)	API release rate in rest	= krel _b_	= mouse	= mouse	= mouse	
*v*_*liver*_	Liver vascular volume fraction	0.125	= mouse	= mouse	= mouse	
*v*_*spleen*_	Spleen vascular volume fraction	0.016	= mouse	= mouse	= mouse	

*Davies et al [5]

+ Ν_ΒR_ was set to zero based on previous assessments [12], since if it was allowed to float, obtained value approximately equal to zero.

++ KNBS was set to 1000 based on previous model fits [12] showing that if parameter allowed to float maximum allowable value was reached. Any distribution rate from spleen back to blood is effectively removed therefore it was deemed reasonable to set KNBS to 1000.

Total API concentrations were obtained by adding released and nanoparticle-conjugated API concentrations for each compartment. Total API concentrations for the blood (*C*_*blood*,*tot*_), plasma(*C*_*plasma*,*tot*_), liver (*C*_*liver*,*tot*_), and spleen (*C*_*spleen*,*tot*_) were captured as follows:

$$C_{blood,tot}=C_{blood,released}+\frac{X_{b\;}}{V_{Nb}}$$ (18)

$$C_{plasma,tot}=C_{plasma,released}+\frac{X_{b\;}}{V_{Nb}*(1-H)}$$ (19)

where *H* is the hematocrit.


$$C_{liver,tot}=C_{liver,released}+\frac{X_{L}}{V_{L}}+v_{Liver}*C_{blood,tot}$$ (20)

$$C_{spleen,tot}=C_{spleen,released}+\frac{X_{s}}{V_{S}}+v_{Spleen}*C_{blood,tot}$$ (21)

Where *v*_*liver*,_ *v*_*spleen*_ are the vascular volume fractions of relevant tissues.

#### Model parameters across species

The model explored within this manuscript was originally fit to mouse data and subsequently scaled to rat and dog. A subset of parameters–such as blood, liver and spleen volumes (*V*_*b*_, *V*_*L*_, *V*_*s*_) and their respective blood flow rates (*Q*_*BL*_, *Q*_*BS*_)–have been well studied across species and were set to their physiological values as reported in the literature [5]. Other parameters, such as API release rates (*krel*_*b*_, *krel*_*L*_, krel_S_) and vascular volume fractions across tissues (*v*_*liver*_, *v*_*spleen*_), were maintained across species, given the lack of evidence for cross-species differentiation. The remaining subset of parameters were allometrically scaled from mouse to rat or dog to account for species differences. These parameters included the API central and peripheral compartment volumes (*V*_*b*_, *V*_*R*_), intercompartmental clearance of the API (Q_BR_), partition coefficients for various compartments and extravasation rates of the nanoparticle-conjugated API, discussed in detail below.

The API central and peripheral compartment volumes (*V*_*b*_ and *V*_*R*_, respectively) were scaled from mouse to rat or dog by correcting for blood protein binding between species [35]:

$${{{[V}_{x}]}_{species\;}={[V}_{x}]}_{mouse\;}*\left[\frac{{fu}_{,blood-species}}{{fu}_{,blood-mouse}}\right]$$ (22)

Where Vx represents volume of either blood (subscript ‘x’ = b) or rest (subscript ‘x’ = R)

The flow rate from blood to “rest” (*Q*_*BR*_) was scaled using the standard power-law equation, based on the bodyweight (*BW*) ratio of the two species and using a standard exponent of 0.7 [36]. Note that the implementation of the allometric relationship, requires for the flow rate to be in units of L/h.


$${\left[Q_{BR}\right]}_{species}=\frac{(\;{\left[\;Q_{BR}\right]}_{mouse\;}*{BW}_{mouse}){\left[\frac{{BW}_{species}}{{BW}_{mouse}}\right]}^{0.7}}{{BW}_{species}}$$ (23)

Partition coefficients of the API in liver and spleen were saturable as shown in Eqs 12 and 13. Parameters descriptive of non-specific binding (*P*_*L*_, *P*_*S*_) and the maximum binding capacity in spleen and liver (*Bmax*_*L*_, Bmax_*S*_) were allometrically scaled, using blood protein binding as a correction factor, as shown below. The partition coefficient of the nanoparticle-conjugated API (*K*_*NBL*_) was also scaled using the following relationship:

$${K_{species\;}=K}_{mouse\;}*\left[\frac{{fu}_{,bloodspecies}}{{fu}_{,blood\;mouse}}\right]$$ (24)

K represents any of the following parameters: *P*_*L*_, *P*_*S*_, *Bmax*_*L*_, Bmax_*S*_ and *K*_*NB*_.

It should be noted that the dissociation constants in liver and spleen, *KD*_*L*_ and K*D*_*S*_, were considered constant across species.

The extravasation rates of the nanoparticle-conjugated API in liver and spleen (*N*_*BL*,_ *N*_*BS*_), were perceived as fractions of the blood flow to the organs, and were therefore scaled based on the blood-to-liver (*Q*_*BL*,*i*_) or blood-to-spleen (*Q*_*BS*_) flow rate ratios between species:

$${\left[N_{BX}\right]}_{species}={\left[N_{BX}\right]}_{mouse\;}*\;\left[\frac{{Q_{BX}}_{species}}{{Q_{BX}}_{mouse}}\right]$$ (25)

where N_BX_ represents the extravasation rate from blood to either liver (subscript ‘x’ = L) or spleen (subscript ‘x’ = S); Q_BX_ represents the blood flow rate to liver (subscript ‘x’ = L,i) or spleen ((subscript ‘x’ = S).

## Supporting information

S1 File (DOCX)

S2 File (M)

S3 File (M)
