# Supplementary material for: Interspecies evaluation of a physiologically based pharmacokinetic model to predict the biodistribution dynamics of dendritic nanoparticles
Source: PLoS One. 2023 May 17;18(5):e0285798. doi: 10.1371/journal.pone.0285798 (PMC10191279; doi:10.1371/journal.pone.0285798)
Supplement: S1 File — (DOCX) [file pone.0285798.s001.docx]

# Supplemental Figures


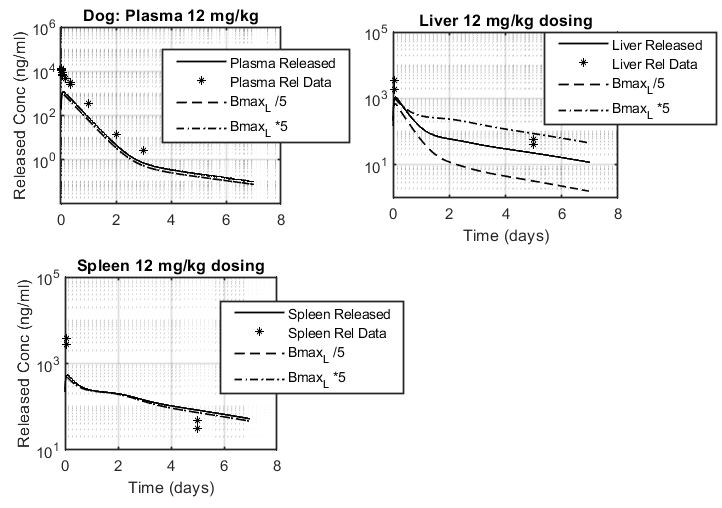


Figure S1: Released API concentration levels in dog as a function of the Bmax_L_ . Data are represented in asterisks; simulated released API profiles are shown in solid line for nominal Bmax_L_ value, dashed line for 5-fold reduction in Bmax_L_ and dashed-dotted line for 5-fold increase Bmax_L_.


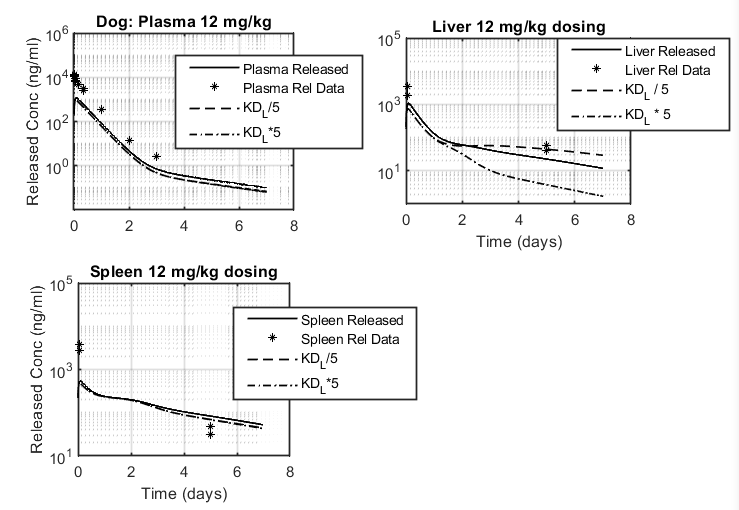


Figure S2: Released API concentration levels in dog as a function of the KD_L_ . Data are represented in asterisks; simulated released API profiles are shown in solid line for nominal KD_L_ value, dashed line for 5-fold reduction in KD_L_ and dashed-dotted line for 5-fold increase KD_L_.


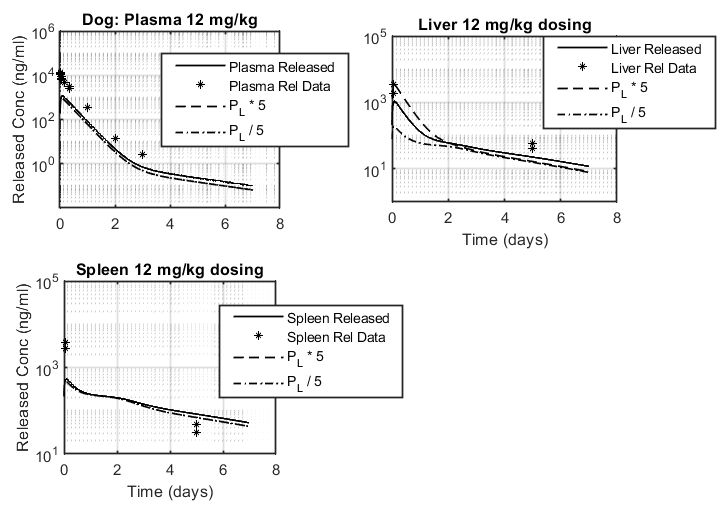


Figure S3: Released API concentration levels in dog as a function of the P_L_. Data are represented in asterisks; simulated released API profiles are shown in solid line for nominal P_L_ value, dashed line for 5-fold reduction in P_L_ and dashed-dotted line for 5-fold increase P_L._

Table S1: Bioavailability estimates of released API

| Species | Dose of API administered as NP (mg/kg) | Plasma AUC_released_API_  (ug/ml*h)  (as shown in Table 1) | Dose of API administered in conventional formulation | Plasma AUC unconjugated API (ug/ml*h)  (as shown in Table 2) | % Bioavailability |
| --- | --- | --- | --- | --- | --- |
| Mouse | 10 | 38.7 | 10 | 5.6 | 692 |
| Rat | 55 | 373 | 50 | 28 | 1211 |
| Rat | 110 | 692 |  |  | 1123 |
| Rat | 505 | 1702 |  |  | 595 |
| Dog | 12 | 53.4 | 12 | 8.9 | 616 |

The bioavailability is calculated as the ratio of dose normalized AUC of NP-released API divided by the dose normalized AUC of the API dosed intravenously in conventional formulation

$$Bioavailability =\frac{\left\lceil\frac{{AUC}_{released_{API}}}{Dose administered as NP} \right\rceil}{}\left\lceil\frac{{AUC}_{API}}{Dose API in conventional formuatlion} \right\rceil$$

# Unconjugated API PK data in mouse, rat and dog

Table S2: Mouse concentrations in ng/ml upon dosing of the unconjugated API as an IV bolus at 10 mg/kg (matching plots in Figure 3)

| Time (hr) | Mouse 1 | Mouse 2 | Mouse 3 |
| --- | --- | --- | --- |
| 0.033333 | 12906.2 | 53776.0 | 32467.3 |
| 0.083333 | 6674.9 | 5317.1 | 5478.4 |
| 0.25 | 2231.7 | 1613.3 | 1942.7 |
| 0.75 | 847.0 | 692.4 | 678.9 |
| 2 | 526.3 | 486.0 | 352.9 |
| 4 | 49.9 | 123.0 | 119.7 |
| 7 | 9.9 | 15.7 | 11.9 |

Table S3: Rat concentrations in ng/ml upon dosing of the unconjugated API as a 30 min infusion at 50 mg/kg (matching plots in Figure 3)

| Time (hr) | Rat 1 | Rat 2 |
| --- | --- | --- |
| 0.5 | 64900 | 64100 |
| 0.75 | 8360 | 10300 |
| 1 | 2220 | 5120 |
| 1.5 | 989 | 1480 |
| 2 | 751 | 662 |
| 4 | 264 | 265 |
| 5 | 111 | 171 |
| 6 | 83.8 | 99.9 |
| 24 | 2.3 | 2.6 |

Table S4: Dog concentrations in ng/ml upon dosing of the unconjugated API as a 3 hr infusion at 12 mg/kg (matching plots in Figure 3)

| Time (hr) | Dog 1 | Dog 2 | Dog 3 | Dog 4 | Dog 5 |
| --- | --- | --- | --- | --- | --- |
| 0.25 | 1492.3 | 2177.9 | 1989.7 | 1976.3 | 2278.8 |
| 3 | 2258.6 | 2043.5 | 2702.2 | 2225.0 | 4503.7 |
| 4 | 93.4 | 75.3 | 80.0 | 132.4 | 383.2 |
| 6 | 25.6 | 20.3 | 20.2 | 38.4 | 301.8 |
| 8 | 10.6 | 8.3 | 7.3 | 13.0 | 154.6 |
| 12 | 2.5 | 2.1 | 1.8 | 3.9 | 37.5 |

# Nanoparticle PK data in mouse, rat and dog

| Time | Total Plasma | Total Tumor | Total Liver | Total Spleen | Released Plasma | Released Tumor | Released Liver | Released Spleen |
| --- | --- | --- | --- | --- | --- | --- | --- | --- |
| 0.0 | 0.0 | 0.0 | 0.0 | 0.0 | 0.0 | 0.0 | 0.0 | 0.0 |
| 0.3 | 436800.0 | 899.7 | 8614.0 | 5889.5 | 3615.4 | 33.9 | 652.7 | 241.9 |
| 0.3 | 376320.0 | 1145.0 | 12100.1 | 7146.9 | 3447.4 | 41.4 | 188.8 | 351.7 |
| 0.3 | 369600.0 | 1332.7 | 11771.2 | 9371.2 | 5510.4 | 47.0 | 680.9 | 380.4 |
| 1.0 | 245280.0 | 1443.1 | 9203.6 | 5615.5 | 3252.5 | 131.8 | 352.4 | 198.3 |
| 1.0 | 282912.0 | 2372.0 | 10973.8 | 6009.6 | 3319.7 | 208.0 | 226.0 | 220.4 |
| 1.0 | 237216 | 1950 | 9978 | 7161 | 3152 | 93 | 511 | 261 |
| 6.0 | 75264 | 4630 | 11693 |  | 4045 | 1062 | 1714 |  |
| 6.0 | 74592 | 9677 | 13843 |  | 4744 | 1068 | 914 |  |
| 6.0 | 8736 | 9341 | 11827 |  | 4032 | 585 | 186 |  |
| 6.0 | 81984 | 4873 | 5846 | 4368 | 1720 | 734 | 196 | 194 |
| 6.0 | 101472 | 5299 | 8089 | 5677 | 1660 | 831 | 384 | 211 |
| 6.0 | 122976 | 5089 | 8472 | 5472 | 2251 | 936 | 470 | 196 |
| 24.0 | 55843 | 6424 | 6337 |  | 269 | 638 | 62 |  |
| 24.0 | 8803 | 3454 | 2634 |  | 177 | 470 | 55 |  |
| 24.0 | 7862 | 4899 | 5087 |  | 161 | 411 | 65 |  |
| 24.0 | 4717 | 1760 | 2363 | 2745 | 362 | 473 | 60 | 47 |
| 24.0 | 6048 | 2684 | 4656 | 3775 | 135 | 586 | 178 | 120 |
| 24.0 | 4906 | 2078 | 3815 | 5157 | 183 | 531 | 149 | 87 |
| 48.0 | 535 | 2769 | 1707 |  | 27 | 161 | 57 |  |
| 48.0 | 381 |  | 3071 |  | 20 |  | 37 |  |
| 48.0 | 574 | 2070 | 1317 |  | 13 | 528 | 83 |  |
| 48.0 | 307 | 1403 | 1598 | 2230 | 11 | 130 | 61 | 89 |
| 48.0 | 159 | 1777 | 1521 | 1849 | 4 | 515 | 42 | 73 |
| 48.0 | 214 | 1740 | 1734 | 2122 | 7 | 198 | 48 | 71 |
| 72.0 | 87 | 778 | 1119 | 1842 | 3 | 53 | 29 | 79 |
| 72.0 | 86 | 1414 | 1713 | 3621 | 2 | 136 | 57 | 162 |
| 72.0 | 75 | 977 | 1040 | 2152 | 2 | 130 | 48 | 85 |
| 96.0 | 71 | 513 | 538 | 1579 | 2 | 30 | 75 | 65 |
| 96.0 | 118 | 744 | 988 | 3313 | 2 | 52 | 18 | 168 |
| 96.0 | 99 | 541 | 734 | 1774 | 1 | 38 | 36 | 74 |

Table S5: Mouse concentrations of total and released API in ng/ml upon dosing of the nanoparticle at 10 mg/kg

Table S6: Rat concentrations of total and released API in ng/ml upon dosing of the nanoparticle at 55 mg/kg

| Time | Total Plasma | Total Tumor | Total Liver | Total Spleen | Released Plasma | Released Tumor | Released Liver | Released Spleen |
| --- | --- | --- | --- | --- | --- | --- | --- | --- |
| 0.5 | 551876 |  |  |  | 51491 |  |  |  |
| 1 | 1270458 |  |  |  | 27964 |  |  |  |
| 8 | 619768 |  |  |  | 11024 |  |  |  |
| 24 | 29846 |  | 56734 |  | 4981 |  | 766 |  |
| 72 | 485 |  |  |  | 22 |  |  |  |
| 168 |  |  |  |  | 5 |  |  |  |
| 0.5 | 15998360 |  |  |  | 52700 |  |  |  |
| 1 | 2157762 |  |  |  | 25006 |  |  |  |
| 8 | 685644 |  |  |  | 9545 |  |  |  |
| 24 | 35223 |  | 47390 |  | 4309 |  | 353 |  |
| 72 | 3011 |  |  |  | 29 |  |  |  |
| 168 |  |  |  |  |  |  |  |  |
| 0.5 | 636573 |  |  |  | 53104 |  |  |  |
| 1 | 2628302 |  |  |  | 21040 |  |  |  |
| 8 | 793196 |  |  |  | 11293 |  |  |  |
| 24 | 36366 |  | 32602 |  | 3011 |  | 259 |  |
| 72 | 370 |  |  |  | 19 |  |  |  |
| 168 |  |  |  |  | 7 |  |  |  |

Table S7: Dog concentrations of total and released API in ng/ml upon dosing of the nanoparticle at 12 mg/kg

| Time | Total Plasma | Total Tumor | Total Liver | Total Spleen | Released Plasma | Released Tumor | Released Liver | Released Spleen |
| --- | --- | --- | --- | --- | --- | --- | --- | --- |
| 0.5 | 284341 |  |  |  | 13377 |  |  |  |
| 0.5 | 290390 |  |  |  | 11831 |  |  |  |
| 0.5 | 266191 |  |  |  | 11831 |  |  |  |
| 0.5 | 252747 |  |  |  | 10755 |  |  |  |
| 1 | 271569 |  | 43559 | 19763 | 8671 |  | 3435 | 2736 |
| 1 | 238631 |  | 17612 | 25476 | 6473 |  | 1875 | 3771 |
| 1 | 250058 |  |  |  | 6856 |  |  |  |
| 1 | 231237 |  |  |  | 6924 |  |  |  |
| 4 | 155950 |  |  |  | 4813 |  |  |  |
| 4 | 153934 |  |  |  |  |  |  |  |
| 8 | 93436 |  |  |  | 3092 |  |  |  |
| 8 | 80664 |  |  |  | 2628 |  |  |  |
| 24 | 7058 |  |  |  | 345 |  |  |  |
| 24 | 6594 |  |  |  | 335 |  |  |  |
| 48 | 174 |  |  |  | 13 |  |  |  |
| 48 | 124 |  |  |  | 13 |  |  |  |
| 72 | 23 |  |  |  | 3 |  |  |  |
| 72 | 31 |  |  |  |  |  |  |  |
| 120 | 13 |  | 786 | 448 |  |  | 59 | 49 |
| 120 | 18 |  | 596 | 325 |  |  | 42 | 31 |
